# Detection of Viruses by Multiplex Real-Time Polymerase Chain Reaction in Bronchoalveolar Lavage Fluid of Patients with Nonresponding Community-Acquired Pneumonia

**DOI:** 10.1155/2020/8715756

**Published:** 2020-11-26

**Authors:** Hao Zhang, Yinling Han, Zhangchu Jin, Yinghua Ying, Fen Lan, Huaqiong Huang, Shaobin Wang, Hongwei Zhou, Rong Zhang, Wen Hua, Huahao Shen, Wen Li, Fugui Yan

**Affiliations:** ^1^Key Laboratory of Respiratory Disease of Zhejiang Province, Department of Respiratory and Critical Care Medicine, Second Affiliated Hospital of Zhejiang University School of Medicine, Hangzhou, Zhejiang 310009, China; ^2^Department of Clinical Laboratory Medicine, Second Affiliated Hospital of Zhejiang University, Hangzhou, China

## Abstract

**Background:**

Nonresponding pneumonia is responsible for the most mortality of community-acquired pneumonia (CAP). However, thus far, it is not clear whether viral infection plays an important role in the etiology of nonresponding CAP and whether there is a significant difference in the clinical characteristics between viral and nonviral nonresponding CAP.

**Methods:**

From 2016 to 2019, nonresponding CAP patients were retrospectively enrolled in our study. All patients received bronchoalveolar lavage (BAL) and virus detection in BAL fluid by multiplex real-time polymerase chain reaction (PCR), and clinical, laboratory, and radiographic data were collected.

**Results:**

A total of 43 patients were included. The median age was 62 years, and 65.1% of patients were male. Overall, 20 patients (46.5%) were identified with viral infection. Of these viruses, influenza virus (*n* = 8) and adenovirus (*n* = 7) were more frequently detected, and others included herpes simplex virus, human enterovirus, cytomegalovirus, human coronavirus 229E, rhinovirus, and parainfluenza virus. Compared with nonviral nonresponding CAP, only ground-glass opacity combined with consolidation was a more common imaging manifestation in viral nonresponding CAP. However, no obvious differences were found in clinical and laboratory findings between the presence and the absence of viral infections.

**Conclusions:**

Viral infections were particularly frequent in adults with nonresponding CAP. The ground-glass opacity combined with consolidation was a specific imaging manifestation for viral nonresponding CAP, while the clinical and laboratory data showed no obvious differences between viral and nonviral nonresponding CAP.

## 1. Introduction

In 2016, lower respiratory infections caused more than two million deaths in people of all ages all over the world [1], which made lower respiratory infections the fourth top cause of deaths. As a leading cause of lower respiratory infections, community-acquired pneumonia (CAP) leads to morbidity and mortality throughout the world in all age groups. A clear understanding of likely pathogens constitutes the basis for selection of empirical antimicrobial agents, which is essential for the management of patients with CAP and improving the prognosis of patients [2–4].

The response of CAP may be inadequate despite antimicrobial treatment, resulting in worsening of the symptoms that may lead to the spreading of infection, development of complications, and even death, which was named nonresponding pneumonia [5, 6]. In the past decades, bacteria as main pathogens of CAP was well characterized, and *Streptococcus pneumoniae* was the most common pathogen [1, 7]. But the existence of unusual microorganisms in CAP is an important cause of nonresponding CAP since these microorganisms are not effectively covered by the recommended initial empiric therapy, including virus. Depending on the development of sensitive molecular laboratory diagnostic tests, recent researches have focused on viral CAP by the method of polymerase chain reaction (PCR) [7–11]. It has been shown that multiplex PCR technology is more sensitive for diagnosis of the main viruses that cause CAP compared with conventional methods [12]. A study on etiological detection of 2320 pneumonia patients completed by Jain S [7] in 2015 showed that the detection rate of virus unexpectedly reached 27%, and another CAP-China network study showed that the positive rate of virus in 2336 hospitalized patients with CAP was 39% [13]. However, thus far, it is not clear whether viral infection plays an important role in the etiology of nonresponding CAP and whether there is a significant difference in the clinical characteristics between viral and nonviral nonresponding CAP. On the other hand, most of the researches of viral pneumonia took the samples by oropharyngeal swab, nasopharyngeal swab, and nasopharyngeal washing. However, about 7.1% healthy people have viral colonization in upper respiratory tract [11, 14], and pneumonia patients may be complicated with upper respiratory tract infection at the same time. Therefore, the use of nasopharyngeal swab samples will increase the possibility of false positive diagnosis of viral pneumonia. Here, we conducted a retrospective study to investigate the role of viral infection in nonresponding CAP by testing virus in bronchoalveolar lavage (BAL) fluid with multiplex real-time PCR for guiding treatment.

## 2. Materials and Methods

### 2.1. Patients

From January 2016 to January 2019, all adults (≥18 years of age) who had been admitted to the Second Affiliated hospital of Zhejiang University School of Medicine in China for CAP, underwent BAL, and were tested for respiratory viruses for poor response to empiric antibiotics therapy were enrolled in the retrospective study. Patients with severe immunosuppressive (HIV infection, solid organ transplantation, or neutropenia<1.0×10^9^/L) were excluded. The study was approved by the Institutional Review Board for Human Studies of Second Affiliated Hospital of Zhejiang University School of Medicine (Hangzhou, China).

### 2.2. Definitions

CAP was defined as a community-acquired acute lower respiratory tract infection with one or more of the following symptoms or signs: cough, productive cough, fever, moist crackles, or signs of pulmonary consolidation, alveolar or interstitial infiltrates shown on a chest radiograph or computer tomography scan that was interpreted as pneumonia by the treating physician.

Nonresponding CAP was defined as persistence of a high temperature (>38°C) and/or clinical symptoms after 72 hours of antibiotic treatment, chest radiographic progression of pneumonia (>50% increase of infiltrates along with persistence of high temperature and/or clinical symptoms), empyema, septic shock, and/or the need for mechanical ventilation.

### 2.3. Data Collection

Clinical, radiographic, and laboratory data and short-term outcomes were collected by a trained physician. A bronchoscope examination was implemented for every patient after admitted to the ward of respiratory department for more than 24 hours later. And BAL fluid was collected from a predominant lesion area following a standardized protocol.

### 2.4. Microbiological Evaluation

Microbiological studies included the following: sputum, BAL fluid for Gram staining and culture; BAL fluid for virus detecting by multiplex real-time PCR, consisting of influenza virus A and B, respiratory syncytial virus A and B, adenovirus, human metapneumovirus, parainfluenza virus types 1 to 4, enterovirus, rhinovirus, cytomegalovirus, herpes simplex virus, human coronavirus 229E/NL63, human coronavirus OC43, human coronavirus HKU1, and bocavirus by using a multiplex real-time PCR kit (Jiangsu Uninovo Biological Technology Co., Ltd., Zhejiang, China). The performance of the respiratory virus multiplex real-time PCR kit used in this study has been evaluated in our previous published studies [15].

### 2.5. Criteria to Establish Presence of Respiratory Pathogens

A diagnosis of respiratory viral infection was made if a virus was detected by BAL fluid. A diagnosis of bacterial infection was made if a viral pathogen was not detected, and the following criteria were met: isolation of a respiratory pathogen from purulent sputum (defined as an adequate quality sputum sample with ≥25 leukocytes and ≤10 epithelial cells per ×100 magnification field), BAL fluid, or blood culture. A mixed infection was defined as the presence of both respiratory virus and bacteria, as defined above. Lastly, if no pathogens were detected, based on the tests used in the study protocol, we classified this as “unknown.”

### 2.6. Statistical Analysis

Categorical variables were described using frequencies and percentages, and continuous variables were presented as means and standard deviation or as median and interquartile range for data. The *χ*^2^ test and Fisher exact test were used regarding categorical variables, while Student's *t*-test and Mann–Whitney *U* test were applied in continuous variables, as appropriate. All data were analyzed using a statistical software package (SPSS, version 23.0; SPSS Inc., Chicago, IL).

## 3. Results

### 3.1. Patient Characteristics

Forty-three of nonresponding CAP patients were sampled in this study (shown in Table 1), with a male to female ratio of 13 : 7. The proportion of patients with bad additions (smoking or drinking) was 44.2% and 34.9%, respectively. Considering that the average age of patients was 62 years old, it is likely to discover other chronic diseases from them, 15 of whom had cardiovascular disease, and 7 had diabetes.

### 3.2. Respiratory Pathogens

Forty-three of BAL fluid specimens were obtained from nonresponding CAP patients. 29 patients (67.4%) had respiratory virus or bacteria identified, of which 20 patients (46.5%) had viral infection and 18 patients had bacterial infection (shown in Table 2). Nine patients were identified with a combined viral and bacterial infection.

Among 20 patients with viral infection, the most common microorganisms were influenza A virus (*n* = 8) and adenovirus (*n* = 7). Others contained herpes simplex virus, human enterovirus, cytomegalovirus, human coronavirus 229E, rhinovirus, and parainfluenza virus. Two patients had two viruses isolated: one had human enterovirus plus adenovirus and the other had cytomegalovirus plus adenovirus. And another patient had human enterovirus, adenovirus plus coronavirus 229E detected.

### 3.3. Laboratory Data

As shown in Table 3, patients with nonresponding CAP usually had a fever (38.0 ± 1.1). Chemistry test demonstrated leukocyte, absolute neutrophil number, percentage of neutrophils, lactate dehydrogenase (LDH), C-reactive protein (CRP), and procalcitonin (PCT) levels were detected higher than normal, whereas absolute number of lymphocytes, percentage of lymphocytes, blood oxygen partial pressure, oxygen saturation, and oxygenation index were lower. And there was no difference between the viral infection and nonviral infection groups in these laboratory data.

### 3.4. Radiographic Features

All 43 patients were examined with high resolution computed tomography (HRCT) scan. As shown in Table 4, most patients presented with bilateral diffuse lung infiltrate. 34 patients (79.1%) had bilateral distribution and 36 patients (83.7%) had diffuse distribution. Pleural effusion (*n* = 20), ground-glass opacity combined with consolidation (*n* = 14), and simply ground-glass (*n* = 13) were also the common HRCT findings among nonresponding pneumonia. Others like peribronchial lesion, nodule, and atelectasis could also be detected. Compared with nonviral nonresponding pneumonia, only ground-glass opacity combined with consolidation was a more common imaging manifestation in viral nonresponding pneumonia (*P* < 0.05), while others showed no differences. These data suggested that the ground-glass opacity combined with consolidation was a specific imaging manifestation for viral nonresponding pneumonia.

### 3.5. Outcomes

The average hospitalization period was 16.9 days. There was high mortality (41.9%) for this cohort of nonresponding CAP patients, which was consistent with the fact that the cure rate of nonresponding CAP was low, while the mortality rate showed no significant difference between the viral and nonviral nonresponding pneumonia.

## 4. Discussion

Our study provides three important findings: (1) the viral infections were particularly common in patients with nonresponding CAP; (2) the ground-glass opacity combined with consolidation on HRCT images was a specific imaging manifestation for viral nonresponding CAP; (3) the clinical and laboratory data showed no obvious differences between viral and nonviral nonresponding CAP patients.

In previous studies, the incidence of nonresponsiveness in hospitalized CAP was 10% to 15% [5], and the mortality of nonresponders was always observed in much higher proportion than that of responders, which ranged from 17.3% to 43.7% [16]. And we confirmed that the mortality of nonresponders was high, reaching more than 40% in our study. Nonresponding pneumonia reflects treatment failure, which is a delay to achievement of clinical stability. And more seriously, it may cause the clinical deterioration named as progressive pneumonia, characterized by the situation of acute respiratory failure requiring mechanical ventilation or septic shock within 72 hours of treatment [6, 17, 18]. Therefore, the identification of pathogens in this population as early as possible is if vital importance, and our study suggested that virus infections should be paid attention to in the etiology of nonresponding CAP.

Several factors like the causal microorganisms, the host, and the antibiotics are relevant with nonresponding pneumonia, and to a large extent the reaction to antibiotics depends on the infection factors. Bacteria is a major component of pathogen causing CAP. Pneumonia caused by *Legionella*, *Staphylococcus aureus,* or Gram-negative bacteria prolongs hospitalization period and is more likely to result in death [19, 20]. However, polymicrobial infection especially virus coinfection may call for more attention. A study reported by Cilloniz et al. [21] found there were 15 in 196 patients with CAP infected with both virus and bacteria, almost all of whom experienced treatment failure. Johansson et al. [22] also found viral and bacterial coinfection led to worse clinical outcomes. In our study, nearly half of nonresponders were detected virus positive, some of whom even had multiple virus infections, which may have a profound effect on nonresponding CAP. Additionally, in this cohort of CAP patients, influenza viruses were mostly detected in winter and spring, showing that influenza has obvious seasonal distribution characteristics. However, other viruses did not have such characteristics.

Viral pneumonia is most typical in children, but with advanced technology, more and more adults are identified with viral infection. In adults, the viral infection rate was found about 10–23% in CAP. Some studies even noted that a third of cases were related with viral infection. The percentage increased more when real-time PCR was utilized. Of these cases, influenza virus, rhinovirus, and coronavirus were relatively frequently detected [9, 23], which was consistent with our findings. Viral pneumonia is different from bacterial pneumonia. Virus-infected individuals usually have a normal or lower leukocyte count, lower CRP, and PCT. Chest radiography often shows bilaterally, sole interstitial infiltrates. Above all, they present slow or no response to antibiotic treatment so as to increase the likelihood of nonresponse among CAP. But despite these different characteristics, it is still difficult to identify whether patients have viral infection or not, especially in nonresponders, for it is generally agreed that secondary bacterial infection is common due to probable impairment to immune systems [8, 9, 22, 23], and nearly half of the cases in our study found that infections overlapped with virus and bacteria.

In this study, there was a total increase in leukocytes, neutrophils, CRP, and PCT. And a reduction in blood oxygen partial pressure, oxygen saturation as well as oxygenation index could be detected oppositely, demonstrating that patients were far from reaching a stable state. In nonresponding pneumonia, there was a persistent lung and systemic inflammation. Leukocytes and neutrophils (including both percentage and absolute number) in blood were significantly higher than normal, representing the regulation of inflammation for killing microorganisms, which could modulate inflammatory cytokine response [24]. And in fact, the levels of interleukin- (IL-) 6, IL8, and IL10 were all associated with nonresponsiveness [25, 26]. Some multivariate regression analysis had found that serum levels of CRP and PCT were strong predictors for the severity of pneumonia. Continuous elevated levels or more rapid growth of CRP is closely related to treatment failure [26–29]. Besides, bilateral and diffuse distribution in radiological images could be observed in most nonresponders, nearly 80%. Ground-glass opacity, consolidation and pleural effusion also took a large proportion. Menendez et al. [24] found multilobar infiltration and pleural effusion were independent risk factors for treatment failure. Similarly, our study also demonstrated that most patients presented with bilateral diffuse lung infiltrate. Furthermore, we showed that the ground-glass opacity combined with consolidation was a specific imaging manifestation for viral nonresponding CAP, which may be useful for the diagnosis of viral pneumonia.

Unfortunately, we found no significant differences in serum markers and clinical features between two groups whether infected virus or not. Undoubtedly, symptoms and laboratory and radiological data are only a supplemental tool to support the current clinical state. Pathogenic identification, after all, is golden standard and important basis for effective treatment methods. The samples can be taken by either invasive or noninvasive ways. Sputum, nasopharyngeal/oropharyngeal swabs, and blood samples are common noninvasive detection methods to collect samples for an etiological diagnosis of CAP [30]. However, the respiratory tract is colonized by viral and bacterial pathogens, leading to the difficulty to differentiate commensal organisms from pathogens. A prospective study found respiratory virus like influenza virus and rhinovirus could also be detected, though lower, from upper respiratory tract (URT) among healthy subjects [11]. In addition, viral load may be lower in URT [23] and the risk of contamination is relatively higher. Therefore, BAL, though invasive but safe, shows its superiority in nonresponders for the sensitivity, specificity, and reliability [30–34]. Besides sample collecting methods, the pathogen detection method is also critical. Respiratory virus identification from lots of previous studies relied on the conventional diagnostic methods such as antibody detection and viral culture. But these tests present many limitations in insensitivity, and virus like rhinovirus and coronavirus can only be distinguished by real-time PCR [8, 11, 23]. As a result, the application of multiplex real-time PCR in BAL fluid plays a crucial role in identifying the virus which attacked nonresponders, guiding therapy even if performed during antibiotic treatment.

There are some limitations in this study. First, it is a retrospective study so that the data are limited. Some clinical data like oxygenation index are not available in several nonresponders for the loss of arterial blood gas analysis, and selection bias also follows. Second, the deficient number of cases affects the credibility of the result. Finally, the actual incidence of bacterial etiology might be underestimated for almost all people have received prior antibiotic treatment.

## 5. Conclusions

In conclusion, the proportion of respiratory virus infection is high among patients with nonresponding CAP, and in patients with nonresponding pneumonia, it is difficult to distinguish patients with viral infection or not. Multiplex real-time PCR in BAL fluid is an effective diagnosis approach for respiratory virus identification even when antibiotic therapy has been performed. In terms of treatment, for simple viral nonresponding pneumonia, targeted antiviral treatment should be selected instead of abuse of antibiotics.

## Figures and Tables

**Table 1:** Baseline characteristics of patients with viral and nonviral infections.

Characteristics	All patients (*n* = 43)	Viral infections (*n* = 20)	Nonviral infections (*n* = 23)	*P* value
*Sociodemographic *				
Age, *y*	62.1 ± 13.7	61.5 ± 12.7	62.7 ± 14.7	0.787
Male sex	28 (65.1)	16 (80.0)	12 (52.2)	0.056
Current or past smoker	19 (44.2)	10 (50.0)	9 (39.1)	0.474
Alcohol use	15 (34.9)	8 (40.0)	7 (30.4)	0.512
*Comorbidities *				
Respiratory disease	6 (14.0)	1 (5.0)	5 (21.7)	0.192
Neurologic disease	5 (11.6)	3 (15.0)	2 (8.7)	0.65
Diabetes	7 (16.3)	4 (20.0)	3 (13.0)	0.687
Cardiovascular disease	15 (34.9)	8 (40.0)	7 (30.4)	0.512
Tumor	7 (16.3)	4 (20.0)	3 (13.0)	0.687
*Prognosis*				
Hospitalization period	16.9 ± 10.2	15.0 ± 8.5	18.6 ± 11.3	0.257
Mortality rate	18 (41.9)	7 (35.0)	11 (47.8)	0.395

Data are presented as mean ± standard deviation or *n* (%).

**Table 2:** Bacterial and viral test result.

Item (bacteria†)	Total (*n* = 43)	Item (virus^‡^)	Total (*n* = 43)
*Stenotrophomonas maltophilia*	2	Herpes simplex virus	2
*Staphylococcus aureus*	3	Influenza A virus	8
*Klebsiella pneumoniae*	1	Human enterovirus	2
*Flavobacterium meningitidis*	1	Human coronavirus 229E	1
*Acinetobacter baumannii*	6	Adenovirus	7
*Klebsiella pneumoniae* pneumonia	4	Cytomegalovirus	2
*Escherichia coli*	1	Rhinovirus	1
*Ralstonia*	1	Parainfluenza virus	1
Other bacteria	4	—	—

^†^Five patients had two bacteria isolated. ^‡^Two patients had two viruses isolated. One patient had three viruses isolated.

**Table 3:** Pathological performance.

Item	Total (*n* = 43)	Patients of viral positive (*n* = 20)	Patients of viral negative (*n* = 23)	*P* value	Judgment
Body temperature	38.0 ± 1.1	38.0 ± 1.1	38.0 ± 1.1	0.881	High^§^
Breath rate	20.7 ± 3.4	19.9 ± 2.6	21.4 ± 3.9	0.13	—
Heart rate	94.8 ± 16.0	89.5 ± 15.5	99.4 ± 15.2	0.04	Significant difference between P&N^*∗*^
Systolic blood pressure	123.7 ± 16.4	121.4 ± 14.7	125.7 ± 17.9	0.394	—
Diastolic blood pressure	71.1 ± 10.0	68.4 ± 7.3	73.5 ± 11.4	0.093	—
Leukocyte	9.7 ± 6.1	8.6 ± 6.5	10.7 ± 5.7	0.271	High^§^
Absolute number of neutrophils	8.4 ± 5.9	7.5 ± 6.2	9.2 ± 5.6	0.37	High^§^
Percentage of neutrophils	83.6 ± 10.0	85.5 ± 8.8	82.0 ± 10.8	0.257	High^§^
Absolute number of lymphocytes	0.75 ± 0.5	0.6 ± 0.5	0.8 ± 0.4	0.207	Low^§^
Percentage of lymphocytes	9.7 ± 6.4	9.2 ± 5.6	10.1 ± 7.0	0.638	Low^§^
Platelet	218.2 ± 119.6	212.7 ± 148.8	223.0 ± 90.1	0.78	—
Lactate dehydrogenase	371 (231–520)	374 (227.3–665.5)	371 (247–460)	0.789	High^§^
C-reactive protein	124.3 ± 79.1	134.8 ± 84.6	115.0 ± 74.5	0.42	High^§^
Urea nitrogen	7.5 ± 7.8	6.7 ± 3.6	8.1 ± 10.2	0.584	—
Creatinine	55 (45–76)	63 (45.5–79)	50 (41–71)	0.342	—
BMI	22.7 ± 3.5	23.0 ± 3.0	22.6 ± 3.8	0.687	—
Procalcitonin	1.3 ± 3.0	1.7 ± 3.4	1.0 ± 2.8	0.467	High^§^
Blood oxygen partia pressure	81.5 ± 25.5	87.0 ± 30.4	76.9 ± 20.2	0.246	Low^§^
Oxygen saturation	94.3 ± 8.3	96.0 ± 2.6	92.9 ± 11.0	0.275	Low^§^
Oxygenation index	249.4 ± 117.6	200.3 ± 126.3	306.7 ± 82.0	0.105	Low^§^

Data are presented as mean ± standard deviation or median (interquartile range). §Judgment refers to the comparison of nonresponding cases versus normal subjects. ^*∗*^Significant difference between patients of viral positive and patients of viral negative with nonresponding CAP.

**Table 4:** Pulmonary CT result.

Item	Total (*n* = 43)	Patient of viral positive (*n* = 20)	Patient of viral negative (*n* = 23)	*P* value
Bilateral distribution	34 (79.1)	15 (75)	19 (82.6)	0.711
Diffuse distribution	36 (83.7)	17 (85)	19 (82.6)	1
Ground-glass	13 (30.2)	5 (25)	8 (34.8)	0.486
Consolidation	6 (14)	1 (5)	5 (21.7)	0.192
Peribronchial lesion	1 (2.3)	0 (0)	1 (4.3)	1
Nodule	2 (4.7)	1 (5)	1 (4.3)	1
Ground-glass opacity combined with consolidation	14 (32.6)	10 (50)	4 (17.4)	0.023^*∗*^
Consolidated nodule	1 (2.3)	1 (5)	0 (0)	0.465
Peribronchial lesions with nodules	4 (9.3)	2 (10)	2 (8.7)	1
Ground-glass opacity combined with nodules	2 (4.7)	1 (5)	1 (4.3)	1
Pleural effusion	20 (46.5)	7 (35)	13 (56.5)	0.158
Atelectasis	1 (2.3)	0 (0)	1 (4.3)	1

Data are presented as *n* (%). ^*∗*^Significant difference between patients of viral positive and patients of viral negative with nonresponding CAP.

## Data Availability

The data used to support the findings of this study are included within the article.

## Additional Points

*Reporting checklist.* We present the article in accordance with the STROBE reporting checklist and the detailed description can be found in the supplementary materials.

## Supplementary Materials

Supplementary Materials STROBE Statement: checklist of items that should be included in reports of cross-sectional studies.
