# Supplementary material for: Detection of Viruses by Multiplex Real-Time Polymerase Chain Reaction in Bronchoalveolar Lavage Fluid of Patients with Nonresponding Community-Acquired Pneumonia
Source: Can Respir J. 2020 Nov 26;2020:8715756. doi: 10.1155/2020/8715756 (PMC7714605; doi:10.1155/2020/8715756)
Supplement: Supplementary Materials — STROBE Statement: checklist of items that should be included in reports of cross-sectional studies. [file 8715756.f1.doc]

STROBE Statement—Checklist of items that should be included in reports of ***cross-sectional studies***

|  | Item No | Recommendation | Reported on Line Number | Reported on Section |
| --- | --- | --- | --- | --- |
| **Title and abstract** | 1 | (*a*) Indicate the study’s design with a commonly used term in the title or the abstract | Line 30 to 33 | Abstract |
|  |  | (*b*) Provide in the abstract an informative and balanced summary of what was done and what was found | Line 34 to 42 | Abstract |
| Introduction | | |  |  |
| Background/rationale | 2 | Explain the scientific background and rationale for the investigation being reported | Line 50 to 57 | Introduction |
| Objectives | 3 | State specific objectives, including any prespecified hypotheses | Line 79 to 81 | Introduction |
| Methods | | |  |  |
| Study design | 4 | Present key elements of study design early in the paper | Line 30 to 33 | Abstract |
| Setting | 5 | Describe the setting, locations, and relevant dates, including periods of recruitment, exposure, follow-up, and data collection | Line 107 to 108 | Methods |
| Participants | 6 | (*a*) Give the eligibility criteria, and the sources and methods of selection of participants | Line 83 to 91 | Methods |
| Variables | 7 | Clearly define all outcomes, exposures, predictors, potential confounders, and effect modifiers. Give diagnostic criteria, if applicable | Line 119 to 127 | Methods |
| Data sources/ measurement | 8* | For each variable of interest, give sources of data and details of methods of assessment (measurement). Describe comparability of assessment methods if there is more than one group | Line 109 to 118 | Methods |
| Bias | 9 | Describe any efforts to address potential sources of bias | Line 87 to 89 | Methods |
| Study size | 10 | Explain how the study size was arrived at | Line 92 to 102 | Methods |
| Quantitative variables | 11 | Explain how quantitative variables were handled in the analyses. If applicable, describe which groupings were chosen and why | Line 119 to 127 | Methods |
| Statistical methods | 12 | (*a*) Describe all statistical methods, including those used to control for confounding | Line 128 to 134 | Methods |
|  |  | (*b*) Describe any methods used to examine subgroups and interactions | - | - |
|  |  | (*c*) Explain how missing data were addressed | - | - |
|  |  | (*d*) If applicable, describe analytical methods taking account of sampling strategy | - | - |
|  |  | (*e*) Describe any sensitivity analyses | - | - |
| Results | | |  |  |
| Participants | 13* | (a) Report numbers of individuals at each stage of study—eg numbers potentially eligible, examined for eligibility, confirmed eligible, included in the study, completing follow-up, and analysed | Line 143 to 146 | Results |
|  |  | (b) Give reasons for non-participation at each stage | Line 87 to 89 | Methods |
|  |  | (c) Consider use of a flow diagram | - | - |
| Descriptive data | 14* | (a) Give characteristics of study participants (eg demographic, clinical, social) and information on exposures and potential confounders | Line 137 to 141 | Results |
|  |  | (b) Indicate number of participants with missing data for each variable of interest | - | - |
| Outcome data | 15* | Report numbers of outcome events or summary measures | Line 175 to 178 | Results |
| Main results | 16 | (*a*) Give unadjusted estimates and, if applicable, confounder-adjusted estimates and their precision (eg, 95% confidence interval). Make clear which confounders were adjusted for and why they were included | - | - |
|  |  | (*b*) Report category boundaries when continuous variables were categorized | Line 154 to 160 | Results |
|  |  | (*c*) If relevant, consider translating estimates of relative risk into absolute risk for a meaningful time period | - | - |
| Other analyses | 17 | Report other analyses done—eg analyses of subgroups and interactions, and sensitivity analyses | - | - |
| Discussion | | |  |  |
| Key results | 18 | Summarise key results with reference to study objectives | Line 180 to 184 | Discussion |
| Limitations | 19 | Discuss limitations of the study, taking into account sources of potential bias or imprecision. Discuss both direction and magnitude of any potential bias | Line 259 to 264 | Discussion |
| Interpretation | 20 | Give a cautious overall interpretation of results considering objectives, limitations, multiplicity of analyses, results from similar studies, and other relevant evidence | Line 256 to 258 | Discussion |
| Generalisability | 21 | Discuss the generalisability (external validity) of the study results | Line 266 to 270 | Conclusions |
| Other information | | |  |  |
| Funding | 22 | Give the source of funding and the role of the funders for the present study and, if applicable, for the original study on which the present article is based | Line 374 to 375 | Footnote |

*Give information separately for exposed and unexposed groups.

**Note:** An Explanation and Elaboration article discusses each checklist item and gives methodological background and published examples of transparent reporting. The STROBE checklist is best used in conjunction with this article (freely available on the Web sites of PLoS Medicine at http://www.plosmedicine.org/, Annals of Internal Medicine at http://www.annals.org/, and Epidemiology at http://www.epidem.com/). Information on the STROBE Initiative is available at www.strobe-statement.org.
